# Blood pressure reactivity at onset of mental stress determines sympathetic vascular response in young adults

**DOI:** 10.14814/phy2.13944

**Published:** 2018-12-14

**Authors:** Khadigeh El Sayed, Vaughan G. Macefield, Sarah L Hissen, Michael J Joyner, Chloe E. Taylor

**Affiliations:** ^1^ School of Medicine Western Sydney University Sydney New South Wales Australia; ^2^ Neuroscience Research Australia Sydney New South Wales Australia; ^3^ Baker Heart and Diabetes Institute Melbourne Victoria Australia; ^4^ School of Science and Health Western Sydney University Sydney New South Wales Australia; ^5^ Department of Anesthesiology Mayo Clinic Rochester Minnesota

**Keywords:** Baroreflex sensitivity, mental stress, muscle sympathetic nerve activity, sex differences

## Abstract

We have previously shown in young males that the rate of rise in blood pressure (BP) at the onset of mental stress determines whether or not muscle sympathetic nerve activity (MSNA) has a role in driving the pressor response. The aim of this study was to investigate these interactions in young females. BP and MSNA were recorded continuously in 19 females and 21 males during 2‐min mental stressors (mental arithmetic and Stroop test). Physical stressor tasks (cold pressor, handgrip exercise, postexercise ischemia) were also performed. During the first minute of mental arithmetic, the rate of rise in mean arterial pressure (MAP) was significantly greater in negative responders (mean decrease in MSNA) compared with positive responders (mean increase in MSNA) in both males (1.9 ± 0.7 vs. 0.7 ± 0.3 mmHg/sec) and females (1.0 ± 0.3 vs. 0.5 ± 0.2 mmHg/sec). For the Stroop test, there was no significant difference in the rate of the rise in BP between positive and negative responders (*P* > 0.05). However, peak changes in MAP were significantly greater in negative responders compared with positive responders in both males (22 ± 6 vs. 13 ± 3 mmHg) and females (12 ± 2 vs. 6 ± 1 mmHg). Sympathetic baroreflex sensitivity was greater in negative responders and may contribute to the fall in MSNA experienced by these individuals during mental stress. During physical stressors there were consistent increases in BP and MSNA in males and females. The findings suggest that, in both males and females, BP reactivity at the onset of mental stress dictates whether or not there is an increase or decrease in MSNA.

## Introduction

Fundamental sex differences in arterial pressure regulation have previously been demonstrated (Shoemaker et al. 2001; Charkoudian et al. 2005; Carter and Ray 2009). Charkoudian et al. (2005) reported that in young healthy men, total peripheral resistance (TPR) is positively related to muscle sympathetic nerve activity (MSNA), suggesting that MSNA is a good index of net vasoconstrictor drive to systemic blood vessels. Hart et al. (2009) reported that high levels of MSNA and high TPR are balanced by lower cardiac output and decreased vasoconstrictor responsiveness to adrenergic stimuli in young men. However, in young women, MSNA is not correlated with either TPR or cardiac output, thus indicating that young women regulate blood pressure differently. Moreover, other studies suggest that there are distinct sex differences in sympathetic control when the human body is exposed to a physiological challenge, such as responding to gravitational load during changes in posture. Convertino (1998) and Barnett et al. (1999) have shown that there are lower plasma noradrenaline concentrations in women during orthostatic stress than in men, suggesting that sympathetic vasoconstrictor outflow may also be diminished.

In studies of mental stress, larger systolic blood pressure responses have been reported in young males compared with young females, despite lower perceived stress scores and similar changes in MSNA and heart rate (Carter and Ray 2009). Whilst MSNA has a clear role in driving increases in blood pressure during physical tasks such as handgrip exercise and the cold pressor test, the interactions between blood pressure and MSNA during mental stress appear to be more complex. We have previously demonstrated in young males that, despite consistent elevations in blood pressure, some individuals experience increases (positive responders) and others decreases in MSNA (negative responders) during mental stress (El Sayed et al. 2016). Interestingly, negative responders exhibited rapid increases in diastolic pressure at the onset of the mental stressor, which lead to reductions in MSNA, presumably due to baroreflex suppression of sympathetic outflow. In positive responders, however, elevations in BP were slower and accompanied by a gradual rise in MSNA, which may have generated or contributed to the pressor response. These results indicate that the blood pressure response at the onset of mental stress dictates whether or not MSNA increases or decreases. Given the evidence supporting sex differences in sympathetic vasoconstrictor control of blood pressure, it is possible that these interactions may differ between males and females.

Briant et al. (2016) have shown that vascular transduction of MSNA is lower in young females than young males, which may lead to a more delayed rise in BP at the onset of mental stress. Based on our previous findings, this reduced rate of rise in BP may lead to reduced baroreceptor stimulation and hence a lower baroreflex‐mediated suppression of MSNA, thus resulting in greater sympathetic activation during the stressor. Christou et al. (2006) have shown that autonomic support of BP and baroreflex buffering is lower in young females than males, which provides further support for the hypothesis that baroreflex suppression of MSNA is reduced during mental stress in females. Therefore, it is hypothesized that there is a greater proportion of positive responders amongst females than in males, and that this group experiences a more gradual rate of rise in BP compared with negative responders. Accordingly, the focus of this study was to examine the early BP response to stress in female positive and negative responders and thus its potential influence on the direction of change in MSNA. As in our previous study in males (El Sayed et al. 2016), the mental stressor tasks used were mental arithmetic and the Stroop color‐word conflict test. Participants also completed a series of physical stressors: cold pressor test, static handgrip exercise and postexercise ischemia.

## Materials and Methods

### Participants

Twenty‐one males and 19 female participants, aged between 18 and 29 years and with no history of cardiovascular disease, were recruited for the study. The data for the male participants have been presented previously (El Sayed et al. 2016), and are included for comparison with the new data obtained from females. Of these, two out of the 19 participants were taking oral contraceptives. Females were tested during the low hormone phase (between days 1–7) of their menstrual cycle or in the low hormone phase of oral contraceptive use to control for the influence of sex hormones on cardiovascular control (Minson et al. 2000a). All studies were conducted with the approval of the Human Research Ethics committee, Western Sydney University, and satisfied the Declaration of Helsinki. Written informed consent was obtained from each participant and they were informed that they could withdraw from the study at any time. Participants were asked to avoid alcohol, caffeine, and vigorous physical activity for a minimum of 12 h before an experiment.

### Measurements

All participants were seated in a semirecumbent position with their legs supported in the extended position. A tungsten microelectrode (FHC, Bowdoin, ME, USA) was inserted percutaneously at the fibular head and an uninsulated reference microelectrode was inserted subdermally ~1 cm away. Intraneural stimulation (0.01–1.0 mA, 1 Hz, 0.2 msec pulses), delivered to the microelectrode via an isolated stimulator (Stimulus Isolator, ADInstruments, Dunedin, New Zealand) was used to guide the microelectrode tip into a motor fascicle of the nerve. The experiments took place in a temperature‐controlled laboratory (22–23°C), and recordings were made in a quiet environment to avoid any disturbances to the MSNA recordings (Hart et al. 2017). Experimental sessions typically lasted 2–3 h, with it taking between 20 and 60 min to gain a stable MSNA signal.

Multiunit neural activity was amplified (gain 20,000, bandpass 0.3–5.0 kHz) using an isolated amplifier and headstage (NeuroAmpEX, ADInstruments, Dunedin, New Zealand) and stored on a computer (10‐kHz sampling) using a computer‐based data acquisition system (PowerLab 16SP hardware and LabChart 7 software; ADInstruments, Dunedin, New Zealand). A root‐mean‐square (RMS) processed version of the signal was computed with a moving average of 200 msec. Bursts of MSNA possessed clear cardiac rhythmicity, increased with inspiratory apnea, demonstrated baroreflex modulation, and could not be evoked by a brisk sniff or sudden loud shout, thus differentiating them from skin sympathetic activity (Mano et al. 2006; El Sayed et al. 2012).

Continuous blood pressure was recorded noninvasively, via finger pulse plethysmography (Finometer; Finapres Medical System, Amsterdam, the Netherlands). Blood pressure status was confirmed using automated brachial measurements. Electrocardiographic (ECG) activity was recorded with Ag‐AgCl surface electrodes on the chest sampled at 2 kHz, and respiration recorded via a piezoelectric (strain‐gauge) transducer around the chest, sampled at 0.4 kHz (Pneumotrace, UFI, Morro Bay, CA).

### Experimental procedures

Once a stable recording site was achieved with spontaneous bursts of MSNA, the participants were asked to relax, while baseline cardiovascular measurements were recorded for 10 min. Following the initial 10‐min baseline period, participants completed two mental and three physical stressor tasks. The mental tasks were mental arithmetic and a Stroop color–word test, while the physical tasks were static handgrip exercise, postexercise ischemia and a cold pressor test. Each task lasted for 2 min and was performed in a randomized order, with the exception of postexercise ischemia, which immediately followed the handgrip task. All tasks were separated by a minimum of 5 min rest to ensure that all variables were stable before commencing the next task.

#### Mental arithmetic task

Participants were given a random three‐digit starting number and asked to consecutively subtract seven, verbally stating the answer for a period of 2 min. If the participant gave an incorrect answer, they were notified and reminded of the last correct number; if their answer was correct they continued without feedback.

#### Stroop color‐word test

Using an iPad with a Stroop color‐word application, participants were required to respond via touch‐screen with the correct color of the word displayed on the screen (as opposed to the color the word spells out). The number of correct answers was recorded.

#### Cold pressor task

Participants were required to immerse their dominant hand in ice water for a period of 2 min. During the task the participants were asked to record their pain level using a visual analog scale; 0 describes “no pain” while 10 describes “the worst pain imaginable”.

#### Static handgrip exercise

A handgrip dynamometer was calibrated according to each participant's maximal voluntary contraction (MVC). Participants were instructed to grip the handgrip dynamometer at 35% MVC for 2 min. The %MVC was displayed on the computer screen to provide feedback to the participants.

#### Postexercise ischemia

Five seconds prior to cessation of handgrip exercise, a cuff was inflated around the active arm to 200 mmHg in order to occlude blood flow to the contracting muscles. The cuff remained inflated for 2 min while the participant relaxed their arm. The cardiovascular reactivity to postexercise ischemia will be treated as a separate task to handgrip exercise.

### Data analysis

#### Time course of responses to stressors

The Peak Parameters module (LabChart 7, ADInstruments, Dunedin, New Zealand) was used to detect and measure the amplitude of individual bursts of MSNA. The nerve trace was shifted to account for the conduction delay and adjusted for each participant to account for differences in burst latency. The average shift applied was 1.26 ± 0.01 sec. Mean burst amplitude and number of bursts of MSNA per minute (burst frequency) or per 100 heart beats (burst incidence) were determined. Total MSNA was calculated as burst frequency multiplied by the mean burst amplitude. Total MSNA and burst amplitude values were normalized to individual resting values and expressed as a percentage change from rest. These measures were related to the rest period immediately prior to the stressor in order to minimize the effects of any change in the MSNA signal during the experiment. Systolic BP, diastolic BP, mean arterial pressure (MAP) were determined from the blood pressure waveform using the Peak Parameters module. For the mental arithmetic and Stroop tasks, changes in systolic BP, diastolic BP, MAP, heart rate, total MSNA, MSNA burst frequency and MSNA burst amplitude responses were determined across 15‐sec intervals throughout rest (2‐min prestressor), task and recovery (2‐min poststressor) periods. Mean changes in each variable were also determined for each stressor task by comparing to the average of the 2‐min rest period prior to the stressor.

#### Positive versus negative responders

For those stressor tasks in which the direction of the change in MSNA differed between individuals, the participants were divided into groups of “positive” and “negative” responders; those individuals with a mean increase in MSNA burst frequency during the task were classified as positive responders and those with a mean decrease were classified as negative responders. In order to assess whether BP responses to stressors differed between positive and negative responders, the following comparisons were made between groups:

*Peak changes*: The peak changes in systolic BP, DBP and MAP were compared between positive and negative responders during the first minute of the task. The first minute was chosen because evidence suggests that the majority of the increase in BP during mental stress occurs within the first minute, after which it typically plateaus (Dunn and Taylor 2014). Peak change (mmHg) was defined as the highest BP value during the first minute of the task minus the BP value of the first cardiac cycle of the task.
*Time of peak*: The time of the peak changes in systolic BP, diastolic BP, and MAP during the first minute of the task were compared between positive and negative responders. Time of peak (s) was defined as the number of seconds to reach the peak BP from the start of the task.
*Rate of rise*: The rate of rise in systolic BP, diastolic BP and MAP was compared between positive and negative responders. Rate of rise (mmHg/sec) was defined as peak change (mmHg)/time to peak (s).

### Sympathetic baroreflex sensitivity

Sympathetic BRS was assessed in all participants using Ensemble (Elucimed Ltd., Wellington, New Zealand). The 10‐min rest period at the beginning of the experimental protocol was used for quantifying sympathetic BRS via spontaneous methods (Kienbaum et al. 2001; Hissen et al. 2015). For each participant, the diastolic pressure values for each cardiac cycle were assigned to 3 mmHg bins to reduce the influence of respiratory‐related oscillations (Ebert and Cowley 1992; Tzeng et al. 2009). For each bin the corresponding MSNA burst incidence was determined (number of bursts per 100 cardiac cycles). MSNA burst incidence was plotted against the mean DBP for each bin in order to quantify sympathetic BRS via linear regression. A weighting was applied to account for the number of cardiac cycles associated with each bin (Kienbaum et al. 2001). The acceptance level for baroreflex slopes was set at *r* > 0.5 (Hart et al. 2011; Taylor et al. 2015).

### Vascular transduction

Vascular transduction was assessed in all participants using Ensemble (Elucimed Ltd.). It was quantified by assessing the relationship between diastolic BP and the preceding MSNA burst areas using the 10‐min rest period. This approach, described by Briant et al. (2016), involves summating the MSNA burst areas in a two cardiac cycle window for each diastolic pressure, based on a fixed lag of six–eight cardiac cycles. The MSNA burst area was binned into 1% sec bins, and the mean diastolic BP for each corresponding bin was calculated. Diastolic BP was plotted against MSNA burst area, generating a slope that represents vascular transduction (units of mmHg/%sec). The linear regression was weighted according to the number of cardiac cycles per bin. In line with the approach of Briant et al. (2016), slopes were not excluded due to low *r* values because these may simply reflect poor transduction and thus provide valuable information regarding interindividual differences.

### Statistical analysis

Two‐way ANOVAs were performed for systolic blood pressure, diastolic blood pressure, MAP, heart rate, total MSNA, MSNA burst frequency and MSNA burst amplitude to determine the main effects and interactions between “sex” and “time”. For these analyses positive and negative responders were pooled. Next, two‐way between‐groups ANOVAs were performed with “sex” (males vs. females) and “response” (positive vs. negative responders) as independent variables in order to compare the peak changes, time to peak, and rate of rise in systolic, diastolic and MAP between positive and negative responders and between males and females. All statistical analyses were performed using Prism v6.00 for Mac OS X (GraphPad software, San Diego, CA). The alpha level was set at *P* < 0.05. All values are expressed as means and standard error (SE).

## Results

Of the 48 individuals recruited for the study, suitable MSNA recordings were obtained in 21 males and 19 females. All of these participants completed each of the stressor tasks. However, in one female, the MSNA recording deteriorated during the stressors and therefore the results for mental stressors are limited to 18 female participants. Participant characteristics are shown in Table 1. The BMI for the males and females fell into the overweight category (*≥*25 kg/m^2^). However, this can be explained by the physical activity levels and body composition of the participants. The participants exercised regularly (*≥*2 × per week), with most undergoing resistance training. Fat‐free mass was 66 ± 2 (males) and 46 ± 1 kg (females); these values are above the average for healthy, young individuals (Kyle et al. 2001). The trend for higher systolic BP in males did not reach statistical significance (*P* = 0.19). However, diastolic BP and MAP were significantly higher in females, indicating greater pulse pressure in males. Resting MSNA was significantly higher in males when expressed as burst incidence (*P* < 0.05), there was no significant sex difference when expressed as MSNA burst frequency (*P* > 0.05).

**Table 1:** Baseline sympathetic and cardiovascular variables (mean ± SE) for males (*n* = 21) and females (*n* = 19)

Variable	Males	Females
Age (years)	22 ± 0.4	23 ± 0.8
Body mass (kg)	79.4 ± 2.5	69.0 ± 4.4^a^
BMI (Kg/m2)	25 ± 1	25.7 ± 2
Fat‐free mass (kg)	66 ± 2	46 ± 1^a^
Systolic BP (mmHg)	129 ± 4	120 ± 3
Diastolic BP (mmHg)	61 ± 3	75 ± 2^a^
MAP (mmHg)	79 ± 3	90 ± 2^a^
Heart rate (beats/min)	64 ± 2	70 ± 3
MSNA burst frequency (bursts/min)	36 ± 1	35 ± 1
MSNA burst incidence (bursts/100heartbeats)	58 ± 2	51 ± 2^a^
Sympathetic BRS (bursts/100 hb/mmHg)	−2.31 ± 0.18	−2.26 ± 0.33
Vascular transduction (mmHg/% sec)	0.12 ± 0.01	0.10 ± 0.01

BP, blood pressure; MAP, mean arterial pressure; MSNA, muscle sympathetic nerve activity.

a Significantly different from males (*P* < 0.05).

### Mental arithmetic: positive and negative responders

**Figure 1:**
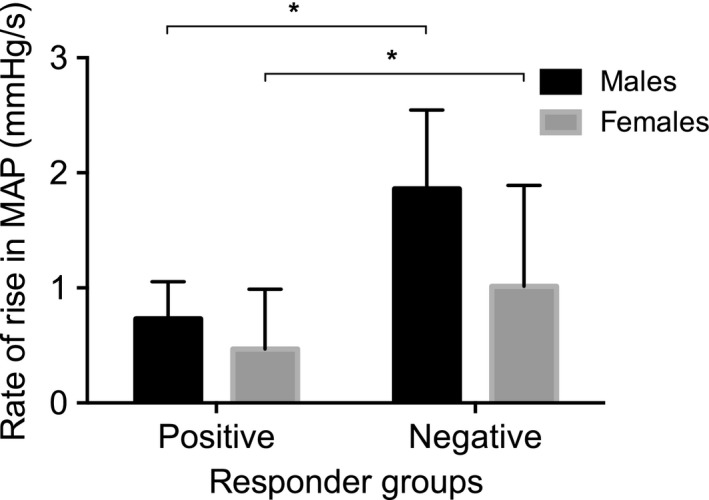
Rate of rise (±SE) in mean arterial pressure (MAP) in male (black bars) and female (gray bars) positive and negative responders to mental arithmetic, *P < 0.05

**Figure 2:**
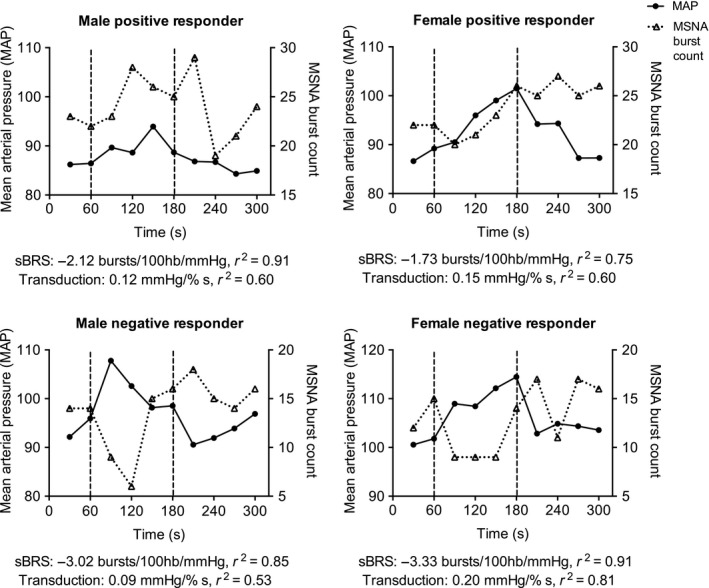
The time course of changes in MAP and MSNA burst frequency during mental arithmetic in two positive responders (one male, one female) and two negative responders (one male, one female). The sympathetic baroreflex sensitivity (sBRS) and vascular transduction values are provided for each individual.

Ten females demonstrated a mean increase in MSNA burst frequency (positive responders) in response to mental arithmetic, and 8 females demonstrated a mean decrease (negative responders). These are similar numbers to those we previously reported in males (13 positive and 8 negative responders) (El Sayed et al. 2016). There were no significant differences in the scores achieved in the mental arithmetic task between positive and negative responders (*P* > 0.05). There were no significant differences in the BP peak changes between responder groups, but there was a significant effect of MSNA response on the time to peak in DBP and MAP, with earlier peaks in negative responders (*P* < 0.05; Table 2). The rate of rise in systolic BP and MAP was significantly greater in negative responders compared with positive responders (Table 2; Fig. 1). Each of these comparisons were consistent between males and females (i.e., no effect of sex, *P* > 0.05). Examples of the time course of changes in MAP and MSNA burst frequency during mental arithmetic are illustrated in Fig. 2, which shows the response in two positive responders (one male, one female) and two negative responders (one male, one female). Typically, negative responders experienced a greater rate of rise in blood pressure early in the task, which was associated with a baroreflex‐driven reduction in MSNA. Positive responders tended to experience a more gradual rise in blood pressure with a concurrent rise in MSNA.

**Table 2:** Peak changes, times to peak and rates of rise in blood pressure (± SE) in positive and negative responders to mental stressor tasks for males (*n* = 21) and females (*n* = 18)

	Systolic BP		Diastolic BP		MAP	
	Males	Females	Males	Females	Males	Females
Peak change (mmHg) for mental arithmetic						
Positive responders	18 ± 3	19 ± 5	10 ± 2	12 ± 3	13 ± 3	14 ± 3
Negative responders	31 ± 7	14 ± 2	13 ± 2	11 ± 3	17 ± 2	11 ± 3
Peak change (mmHg) for Stroop test						
Positive responders	17 ± 4	8 ± 2^b^	10 ± 2	4 ± 1^b^	13 ± 3	6 **±** 1^b^
Negative responders	33 ± 11^a^	15 ± 3^a^, ^b^	23 ± 6^a^	11 ± 2^a^, ^b^	22 ± 6^a^	12 ± 2^a^, ^b^
Time of peak (s) for mental arithmetic						
Positive responders	15 ± 3	36 ± 5	42 ± 6	43 ± 5	43 ± 5	41 ± 4
Negative responders	36 ± 8	24 ± 7	27 ± 8^b^	29 ± 8 a	23 ± 7^a^	24 ± 8 a
Time of peak (s) Stroop test						
Positive responders	39 ± 6	23 ± 7	35 ± 6	14 ± 7	32 ± 6	18 ± 7
Negative responders	39 ± 7	38 ± 7	29 ± 8	33 ± 8	29 ± 8	35 ± 7
Rate of rise (mmHg) for mental arithmetic						
Positive responders	0.7 ± 0.3	0.5 ± 0.1	0.4 ± 0.1	0.9 ± 0.7	0.7 ± 0.3	0.5 ± 0.2
Negative responders	2.2 ± 1.0^a^	2.2 ± 1.6 a	1.3 ± 0.5	0.5 ± 0.2	1.9 ± 0.7^a^	1.0 ± 0.3 a
Rate of rise (mmHg) for Stroop test						
Positive responders	0.9 ± 0.3	1 ± 0.4	0.5 ± 0.2	1.4 ± 0.5	0.9 ± 0.3	1.1 ± 0.5
Negative responders	1.2 ± 0.4	0.8 ± 0.4	1.4 ± 0.4	0.9 ± 0.4	1.3 ± 0.4	0.6 ± 0.3

BP, blood pressure, MAP, mean arterial pressure; MSNA, muscle sympathetic nerve activity.

a Significantly different from positive responders (*P* < 0.05).

b Significantly different from males (*P* < 0.05).

### Mental arithmetic: sex differences

When pooling positive and negative responders, the mental arithmetic task was associated with significant increases in BP, heart rate, total MSNA and MSNA burst amplitude over time (Table 3; *P* < 0.05). There was a significant interaction between time and sex for heart rate (*P* = 0.004), with larger increases in the males than the females. For both sexes the increases in BP occurred *after* the peak in heart rate. There was a significant effect of sex on total MSNA with greater increases in males than females (*P* = 0.02). However, females had greater increases in the recovery period compared with the males. There was an effect of time on MSNA burst frequency but this did not reach significance (*P* = 0.06). Due to the presence of positive and negative responders the mean changes in the MSNA burst frequency during the mental arithmetic task were 0 ± 2 and −3 ± 6 bursts/min for males and females, respectively.

**Table 3:** Mean changes (±SE) in sympathetic and cardiovascular variables during mental stressor tasks for males (*n* = 21) and females (*n* = 18)

Variable	Mental arithmetic		Stroop test	
	Males	Females	Males	Females
Systolic BP (mmHg)	11 ± 3^a^	9 ± 2^a^	8 ± 3^a^	4 ± 2^a^
MAP (mmHg)	14 ± 1^a^	7 ± 1^a^	13 ± 2^a^	4 ± 1^a^
Diastolic BP (mmHg)	5 ± 1^a^	6 ± 1^a^	4 ± 1^a^	3 ± 1^a^
Heart rate (beats/min)	6 ± 2^a^	4 ± 2^a^^,^ c	6 ± 2^a^	4 ± 2^a^
Total MSNA (%)	5 ± 6^a^	2 ± 8^a^^,^ b	0 ± 8	14 ± 9^b^
MSNA burst ampl. (%)	22 ± 15^a^	6 ± 5^a^	5 ± 8	19 ± 13
MSNA burst freq. (bursts min^−1^)	0 ± 2	‐3 ± 6	0 ± 2^a^	0 ± 1^a^

BP, blood pressure; MAP, mean arterial pressure; MSNA, muscle sympathetic nerve activity.

a Significant main effect of time (*P* < 0.05).

b Significantly different from males (*P* < 0.05).

c Significant interaction between time and sex (*P* < 0.05).

### Stroop test: positive and negative responders

**Figure 3:**
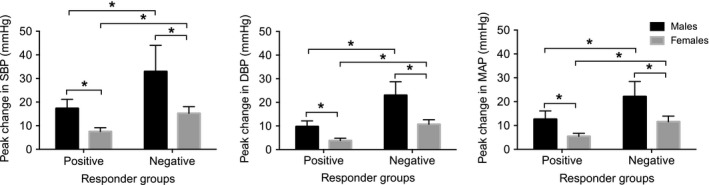
Changes (±SE) in systolic BP, diastolic BP and mean arterial pressure (MAP) in positive and negative responders to the Stroop test in males (black bars) and females (gray bars), * *P* < 0.05. MAP, mean arterial pressure; MSNA, muscle sympathetic nerve activity

For the Stroop test, 9 females demonstrated a mean increase in the MSNA burst frequency (positive responders) and 9 females showed a mean decrease in the MSNA burst frequency (negative responders). Of the 9 positive responders to the Stroop test, 7 were positive responders to mental arithmetic, and of the 9 negative responders to the Stroop test, 6 were negative responders to mental arithmetic. In our previous study in males, we reported 13 positive and 8 negative responders for the Stroop test (El Sayed et al. 2016). There were no significant differences in the scores achieved in the Stroop test between positive and negative responders (*P* > 0.05). There was a significant effect of the MSNA response on the peak changes in systolic BP, diastolic BP and MAP, with greater changes for negative responders compared with positive responders (*P* < 0.05; Table 2; Fig. 3).

### Stroop test: sex differences

When pooling positive and negative responders, the Stroop color–word conflict test was associated with significant mean changes in BP, heart rate, and the MSNA burst frequency during the task (*P* < 0.05; Table 3). There was a significant main effect of time on MSNA burst frequency (*P* = 0.03). However, due to the presence of positive and negative responders the mean change was 0 ± 1 for both males and females. The mean change in total MSNA was 14 ± 9% for females, but there was high interindividual variability in this measure and no significant effect of time (*P* = 0.18). When examining peak changes in BP (Table 2, Fig 3), there were greater peaks in systolic BP, diastolic BP and MAP in males compared with females (*P* < 0.05).

### Sympathetic baroreflex sensitivity

**Figure 4:**
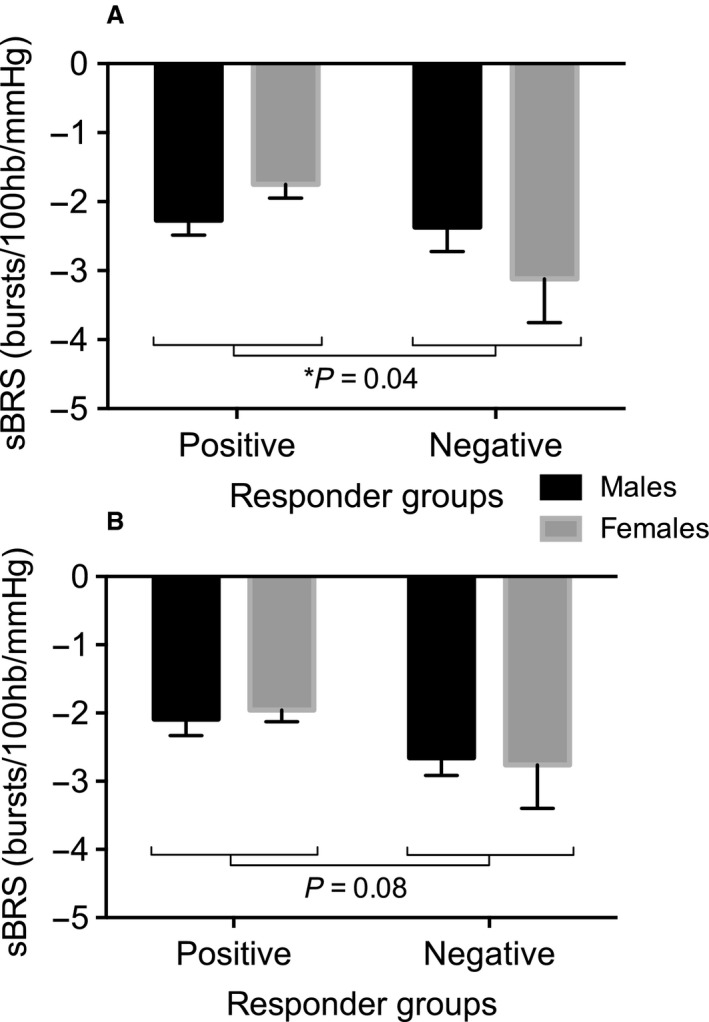
Sympathetic baroreflex sensitivity (sBRS) ±SE in positive and negative responders to mental arithmetic (panel A) and the Stroop test (panel B), * *P* < 0.05.

Sympathetic BRS was significantly greater in negative responders compared with positive responders to mental arithmetic (−2.75 ± 0.36 vs.−2.04 ± 0.16 bursts/100 hb/mmHg; *P* = 0.04). Although there was no significant effect of sex, there was a trend for an interaction between sex and response (*P* = 0.08), with the differences between positive and negative responders being more apparent in females (Fig. 4). There was a trend for greater sympathetic BRS in negative versus positive responders to the Stroop test (−2.71 ± 0.35 vs. 2.04 ± 0.16 bursts/100 hb/mmHg; *P* = 0.08).

### Vascular transduction

**Figure 5:**
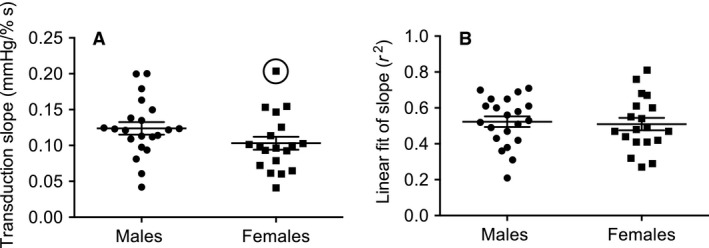
Vascular transduction ± SE (panel A) and *r* ^2^ of linear fit ±SE (panel B) in males and females. Circle represents a potential outlier in the female group.

There were no significant effects of sex or MSNA response on vascular transduction (*P* > 0.05). When positive and negative responders were pooled, vascular transduction was not significantly different between males (0.12 ± 0.01) and females (0.10 ± 0.01 mmHg/%sec, *P* = 0.11), and the linear fit (*r* ^2^) of these transduction slopes did not differ (0.52 ± 0.03 vs. 0.51 ± 0.03, *P* = 0.77; Fig. 5). One female had a particularly high transduction value (0.20 mmHg/%sec, *r* ^2^ = 0.81) compared with the other females. With removal of this potential outlier, sex differences were statistically significant, with greater transduction in males (*P* = 0.03). However, a subsequent MSNA recording from this individual on a different day revealed a similar vascular transduction value and there was no obvious reason to exclude this female. This female was one of two females taking oral contraceptives, but the second female did not have elevated vascular transduction (0.10 mmHg/%sec, *r* ^2^ = 0.55).

### Physical stressors

The cold pressor test caused gradual and concurrent increases in BP and MSNA during the 2‐min task. Significant increases in BP, heart rate, and MSNA occurred (Table 4; *P* < 0.05), but there were no significant differences between males and females (*P* > 0.05). There was no significant difference in peak pain scores between males (6.5 ± 0.5) and females (7.6 ± 0.6; *P* = 0.24). Moreover, linear regression analysis revealed no significant relationship between peak pain score and the mean change in systolic BP for males (*r* ^2 ^= 0.001; *P* = 0.89) or females (*r* ^2 ^= 0.01; *P* = 0.70).

**Table 4:** Mean changes in sympathetic and cardiovascular variables during physical stressor tasks for the males (*n* = 21) females (*n* = 19)

Variable	Cold pressor		Handgrip		Ischemia	
	Males	Females	Males	Females	Males	Females
Systolic BP (mmHg)	18 ± 4 a	19 ± 3^a^	13 ± 3^a^	13 ± 2^a^	14 ± 3^a^	13 ± 4^a^
MAP (mmHg)	11 ± 2^a^	19 ± 2^a^	7 ± 1^a^	11 ± 2^a^, ^c^	5 ± 1^a^	13 ± 3^a^
Diastolic BP (mmHg)	11 ± 2^a^	15 ± 2^a^	11 ± 1^a^	10 ± 2^a^, ^c^	9 ± 2^a^	10 ± 3^a^
Heart rate (beats/min)	6 ± 2^a^	9 ± 2^a^	11 ± 2^a^	4 ± 1^a^^,^ b, c	5 ± 1	‐2 ± 1
Total MSNA (%)	62 ± 11^a^	107 ± 24^a^	34 ± 11^a^	32 ± 17^a^	42 ± 12^a^	56 ± 16^a^
MSNA burst ampl. (%)	30 ± 6^a^	55 ± 15^a^	21 ± 8^a^	19 ± 9^a^	24 ± 6^a^	54 ± 20^a^
MSNA burst freq. (bursts min‐1)	7 ± 2^a^	4 ± 1^a^	3 ± 1^a^	4 ± 1^a^	4 ± 2^a^	3 ± 1^a^

BP, blood pressure; MAP, mean arterial pressure; MSNA, muscle sympathetic nerve activity.

a Significant main effect of time (*P* < 0.05).

b Significantly different from males (*P* < 0.05).

c Significant interaction (*P* < 0.05).

As expected, static handgrip exercise also resulted in significant increases in BP, heart rate, and MSNA over time (Table 4; *P* < 0.05). There was a significant interaction between time and sex for MAP (*P* = 0.003), diastolic BP (*P* = 0.046) and heart rate (*P* = 0.0001), and a significant effect of sex on heart rate (*P* = 0.0142), with greater increases in males compared with females. Males displayed a bigger drop in BP when handgrip exercise ceased, but it still remained significantly elevated above baseline during ischemia for both sexes. In addition, postexercise ischemia was associated with significant increases in BP and MSNA over time (Table 4; *P* < 0.05). There were no significant effects of sex or interaction between time and sex for any of the variables during postexercise ischemia (*P* > 0.05). Since the cold pressor, handgrip and ischemia tasks elicited consistent increases in MSNA between participants, no analyses on positive and negative responders were performed.

## Discussion

In this study, changes in blood pressure during mental stress in males and females were compared between positive and negative responders, that is, those with an overall increase or decrease in MSNA during stress. Consistent with our previous study in young males, female *negative* responders to mental arithmetic exhibited greater blood pressure reactivity at the onset of the stressor, suggesting a baroreflex‐mediated suppression of MSNA. Furthermore, these negative responders also possessed greater sympathetic baroreflex sensitivity than positive responders. As we have previously shown in males, female *positive* responders also exhibit a more gradual rise in blood pressure during mental arithmetic, which appears to be MSNA‐driven. This study indicates that in healthy young adults, regardless of sex, the reactivity of blood pressure early in the task determines whether or not MSNA has a role in driving the pressor response. Sympathetic baroreflex sensitivity, but not vascular transduction, may also play a role in the direction of change in MSNA. Physical stressors such as the cold pressor test, handgrip exercise and postexercise ischemia, are associated with consistent increases in MSNA, parallel to those of BP, in both males and females.

### The role of the baroreflex in MSNA responses to mental stress

The initial blood pressure response to mental stress appears to dictate baroreceptor engagement and the subsequent response. A rapid rise in pressure loads the baroreceptors and drives inhibition of MSNA, whereas a gradual rise in pressure allows the baroreflex to be reset, enabling MSNA to become elevated. We speculated that there is less suppression of MSNA in females throughout the mental tasks, given previous reports of reduced baroreflex buffering of rising blood pressures (Christou et al. 2006). We therefore hypothesized that the rate of rise in blood pressure is smaller in females than males, and thus a larger proportion of females present as positive responders (increase in MSNA) to mental stress. However, we found similar numbers of positive and negative responders in the female group, comparable to the numbers in the male group. Furthermore, the differences in initial blood pressure reactivity between negative and positive responders were present in both sexes and there was no significant difference in sympathetic BRS between males and females.

Importantly, sympathetic BRS was significantly greater in negative responders compared with positive responders. In our previous study in males, we found no significant differences in sympathetic BRS between responder groups (El Sayed et al. 2016). However, the addition of females appears to have provided sufficient statistical power to detect such differences, albeit a subtle difference of approximately 0.7 bursts/100 hb/mmHg. There is currently a lack of consensus in the literature regarding meaningful differences in sympathetic BRS. In intervention studies, changes in sympathetic BRS have been reported in the region of 1.5–2.9 bursts/100 hb/mmHg (Keller et al. 2006; Young et al. 2010; Hart et al. 2013). In studies of sympathetic BRS between groups, differences of 0.99 bursts/100 hb/mmHg have been reported between elderly men and women (Okada et al. 2012), and differences of 2.91 bursts/100 hb/mmHg have been reported between patients with panic disorder and healthy controls (Lambert et al. 2002). Despite these studies, it is not clear what may constitute the smallest meaningful difference and whether a difference of 0.7 bursts/100 hb/mmHg ought to be sufficient to impact blood pressure and MSNA responses to mental stress.

It is possible that the mildly elevated sympathetic BRS in negative responders contributes to the baroreflex‐driven fall in MSNA during the stressor tasks; we would expect greater inhibition of MSNA bursts for a given rise in pressure in these individuals. However, it should be noted that sympathetic BRS was assessed during the 10‐min baseline period and not during the 2‐min stressor tasks, and it is possible that BRS may differ between rest and during mental stress. Durocher et al. (2011) have previously reported that sympathetic BRS is attenuated during the first 2 min of mental stress, which may facilitate increases in MSNA. It is not known whether there are differences in sympathetic BRS during mental stress in positive and negative responders. We did not examine BRS during stress as it has recently shown that short time intervals, such as 2 min, do not produce BRS values consistent with those of longer intervals, and values tend to be artificially inflated (Hissen et al. 2018; Holwerda et al. 2018a). In future studies, longer periods of stress may allow these comparisons to be made.

Participants in this study were assessed in an upright seated position, in contrast to other studies of mental stress in which participants have been supine (Durocher et al. 2011; Yang et al. 2012). Previous research suggests that supine posture is associated with reduced sympathetic BRS compared with more upright postures (O'Leary et al. 2003; Fu et al. 2006). It is possible that the reduction in resting MSNA associated with supine posture may cause some individuals with very low MSNA to operate around a different point on the baroreflex curve, possibly within nonlinear threshold regions (Incognito et al. 2018). However, with no differences in resting MSNA between positive and negative responders, assessing individuals in the supine position would likely reduce sympathetic BRS in the two groups similarly. An upright seated position is arguably the most appropriate for assessing sympathetic BRS and responses to stress given that this is more representative of posture during the daytime, but differences in posture should be considered when comparing studies.

### Vascular transduction in young males and females

We report no differences in vascular transduction between positive and negative responders, suggesting that interindividual differences in vascular transduction do not play a major part in determining the early blood pressure response to mental stress and the resultant change in MSNA. However, in contrast to recent evidence, we also report no significant sex differences in vascular transduction. Briant et al. (2016) have previously demonstrated that young females exhibit diminished transduction of MSNA relative to their male counterparts. While our transduction values for young males are comparable to those of Briant et al. (2016), the presence of a potential female outlier with high vascular transduction may explain the lack of a statistically significant sex difference. However, when excluding this individual, the mean (±SE) transduction value for females remains at 0.10 (±0.01) mmHg/%s, and is higher than the 0.03 (±0.01) mmHg/%sec reported by Briant et al. (2016). Levels of resting MSNA may explain the differences observed in females between the two studies. When compared with the results reported by Briant et al. (2016) for young females, resting MSNA burst frequency (35 ± 1 vs. 19 ± 2 bursts/min) and MSNA burst incidence (51 ± 2 vs. 31 ± 3 bursts/100 hb) were markedly higher in this study. This may be explained by postural differences; participants in this study were seated in a semirecumbent position with their legs supported in the extended position, whereas in the study by Briant et al. (2016) participants were supine. It has previously been shown that passive tilting of the body from supine to as little as 30° head‐up tilt causes significant elevations in MSNA (Delius et al. 1972). It is possible that low resting MSNA associated with supine posture, particularly in females, may limit the available data with which to quantify transduction.

Differences in resting MSNA may also influence the relative contributions of neurotransmitters. Sympathetic vasoconstriction is mediated by noradrenaline and co‐transmitters neuropeptide‐Y and adenosine triphosphate (ATP) (Burnstock 2009). There is evidence from animal models that changes in the frequency of neuronal firing may alter the contributions of each neurotransmitter, for instance, with the contribution of ATP being greater at low‐discharge frequencies (Haniuda et al. 1997). Early evidence indicates that high discharge frequencies favor neuropeptide‐Y (Pernow 1988), although Bradley et al. (2003) report that it acts almost exclusively as a neuromodulator, potentiating the actions of co‐transmitters, and that its contribution may actually be greater at lower firing frequencies. Although more research is required, it is possible that alterations in the relative contributions of these neurotransmitters according to firing rate may influence the magnitude of the vasoconstrictor response, potentially explaining the differences in vascular transduction between groups with different levels of resting MSNA. Further research is required to determine the impact of these changes in neurotransmitter contributions on vasoconstriction in humans, but it is worth pointing out that resting firing rates of human muscle vasoconstrictor neurones are typically very low (~0.5 Hz), with mean firing rates being lower in individuals with higher levels of MSNA at rest than those with low resting levels (Macefield and Wallin 1999).

The differences in vascular transduction may be associated with the lower number of females taking oral contraceptives in this study (2/19, 11%) versus that of Briant et al. (17/23, 74%). It has been reported that the mid‐luteal phase of the menstrual cycle is associated with greater levels of resting MSNA than the early follicular phase (Minson et al. 2000a; Middlekauff et al. 2012), suggesting that female sex hormones may influence sympathetic outflow. However, studies in women taking oral contraceptives indicate no differences in MSNA between the placebo and high hormones phases of the contraceptive pill (Minson et al. 2000b; Middlekauff et al. 2012). Importantly, when women taking oral contraceptives are compared directly to women with natural menstrual cycles in their respective low hormone phases, there are no differences in resting MSNA (Middlekauff et al. 2012; Harvey et al. 2015). Vascular transduction has yet to be directly compared between these two groups, although evidence suggests that transduction does not differ between low and high hormone phases of the natural menstrual cycle (Minson et al. 2000a). There has yet to be a study in which changes in resting MSNA and/or vascular transduction are examined in women with natural menstrual cycles who start taking oral contraceptives.

In this study, vascular transduction was examined by looking at relationships between MSNA and blood pressure at rest. Future studies involving longer periods of mental stress would allow examination of these beat‐to‐beat relationships during stressor tasks. Yang et al. (2012) examined the mean changes in vascular conductance between a 5‐min rest period and a 5‐min mental arithmetic task in males and females. Although both sexes demonstrated elevated forearm vascular conductance during mental stress, as previously shown (Blair et al. 1959; Barcroft et al. 1960), males exhibited reductions in calf vascular conductance but females demonstrated an increase. The changes in calf vascular conductance in males were negatively correlated with changes in MSNA during mental stress, with no apparent relationship for females. This suggests that vascular transduction of MSNA during mental stress may be greater in males than females. However, these findings are based on mean changes in vascular conductance between rest and stress, during which some individuals experienced a decrease or very little change in MSNA thus making it difficult to ascertain vascular transduction on an individual basis. Using beat‐to‐beat methods for quantifying vascular transduction in individuals during mental stress may provide further insight.

### Future directions

Given that skeletal muscles are highly vascularized, changes in muscle blood flow through changes in sympathetically mediated vasoconstrictor drive can have a big effect on blood pressure. While MSNA appears to be involved in driving the BP responses in positive responders, other factors must be responsible in negative responders and of particular interest is what drives the rapid response at the onset of the stressor in these individuals. Elevated heart rate may contribute for the first 30 sec, but it then reaches a peak whilst BP continues to rise. It is possible that there is an increase in stroke volume leading to elevated cardiac output, or an increase in sympathetic outflow to other vascular beds leading to elevated vascular resistance. The interactions between sympathetic outflow and endothelial function may be worthy of further research in the context of blood pressure responses to mental stress. There is some evidence that nitric oxide (NO) levels are higher in individuals with high resting MSNA and that this NO buffers the blood pressure‐raising effects of the high sympathetic activity. Charkoudian et al. (2006) found that modest doses of the nitric oxide inhibitor, N G‐monomethyl l‐arginine (L‐NMMA), caused greater increases in BP in subjects with high resting MSNA than in those with low levels of MSNA. The influence of such interactions during mental stress are unknown, but it is possible that in some individuals NO buffers the initial rise in blood pressure at the onset of stress. With larger sample sizes it may be possible to explore these mechanisms and also divide individuals into groups of positive, negative and nonresponders, that is, those with a mean change in MSNA burst frequency of <3 bursts/min during mental stress.

Evidence suggests that anxiety disorders may be associated with augmented sympathetic activity during stress when compared with healthy controls (Park et al. 2017; Holwerda et al. 2018b). Park et al. (2017) reported an overall increase in MSNA during mental stress, which was exaggerated in those with post‐traumatic stress disorder (PTSD). The direction of the change in MSNA was not reported on an individual basis, but it is possible that PTSD may be associated with a larger proportion of positive responders to mental stress. Interestingly, Park et al. (2017) reported that PTSD was associated with blunted sympathetic BRS at rest, but further research in populations with such disorders is required to determine whether the elevated MSNA response is also associated with a gradual rise in blood pressure at the onset of a stressor task.

This study indicates the importance of considering the *rate of rise in blood pressure* on baroreceptor loading and not just the absolute blood pressure values when assessing baroreflex function. Traditionally, when sympathetic BRS is assessed in the resting state, levels of MSNA are quantified for specific diastolic pressure bins, each representing an absolute BP range. As diastolic pressure rises, greater inhibition of MSNA occurs and thus the percentage of cardiac cycles associated with an MSNA burst is reduced. However, this approach does not take into account the rate of rise in diastolic pressure and thus an assumption is made that the rate of change in blood pressure does not influence the magnitude of the change in MSNA. If the magnitude of the baroreflex response is driven by the *rate of rise in BP* as well as the *absolute pressure changes*, this may explain why plotting sympathetic baroreflex slopes is often reported as unsuccessful in some individuals. Furthermore, it may explain reports of hysteresis, that is, where there are differences in BRS for falling versus rising pressures. If the rise or fall in pressure is not at the same rate then this may affect the extent to which the baroreceptors are loaded and unloaded. Studinger et al. (2009) employed the modified Oxford method to assess sympathetic BRS and reported that it is greater in response to falling pressures compared with rising pressures. In our experience, the rate of fall in pressure following sodium nitroprusside administration is greater than the rate of rise following phenylephrine. If a more rapid change in pressure leads to greater baroreceptor loading/unloading then this may explain the greater sympathetic BRS during the fall in pressure driven by sodium nitroprusside. However, this does not explain why the opposite effect is observed for cardiac BRS, which is reportedly greater in response to rising pressures compared with falling. There has yet to be a study in which the factors influencing baroreceptor loading have been effectively explored.

## Conclusions

In both males and females, the reactivity of blood pressure at the onset of mental stress dictates the direction of change in MSNA during the task. These data indicate that a rapid rise in pressure causes significant loading of the baroreceptors with a resultant fall in MSNA (negative response). A gradual rise in pressure enables baroreflex resetting without substantial baroreceptor loading, allowing MSNA to become elevated during mental stress (positive response) and potentially contributing to the pressor response. Greater sympathetic baroreflex sensitivity may contribute to the fall in MSNA experienced by negative responders during mental stress.

## Conflict of Interest

The authors declare no conflict of interest.
